# TCAF2 drives glioma cellular migratory/invasion properties through STAT3 signaling

**DOI:** 10.1007/s11010-023-04891-0

**Published:** 2023-11-29

**Authors:** Debo Yun, Jianshen Liang, Xuya Wang, Jikang Fan, Xisen Wang, Jiabo Li, Xiao Ren, Jie Liu, Xiude Ren, Hao Zhang, Guanjie Shang, Wenzhe Jin, Lei Chen, Tao Li, Chen Zhang, Shengping Yu, Xuejun Yang

**Affiliations:** 1 Department of Neurosurgery, Tianjin Medical University General Hospital, Tianjin, China; 2 Laboratory of Neuro-Oncology, Tianjin Neurological Institute, Tianjin, China; 3 Department of Neurosurgery, Nanchong Central Hospital, Nanchong, China; 4 Department of Neurosurgery, Beijing Tsinghua Changgung Hospital, Beijing, China

**Keywords:** Glioma, TCAF2, STAT3 signaling pathway, Migration, Invasion

## Abstract

Glioma is an intracranial tumor characterized by high mortality and recurrence rates. In the present study, the association of TRPM8 channel-associated factor 2 (TCAF2) in glioma was investigated using bioinformatics, showing significant relationships with age, WHO grade, IDH, and 1p/19q status, as well as being an independent predictor of prognosis. Immunohistochemistry of a glioma sample microarray showed markedly increased TCAF2 expression in glioblastoma relative to lower-grade glioma, with elevated expression predominating in the tumor center. Raised TCAF2 levels promote glioma cell migratory/invasion properties through the epithelial-to-mesenchymal transition-like (EMT-like) process, shown by Transwell and scratch assays and western blotting. It was further found that the effects of TCAF2 were mediated by the activation of STAT3. These results suggest that TCAF2 promotes glioma cell migration and invasion, rendering it a potential drug target in glioma therapy.

**Supplementary Information:**

The online version contains supplementary material available at 10.1007/s11010-023-04891-0.

## Introduction

Glioma is the most common malignant central nervous system tumor with high mortality [1]. Diffuse tumors lean toward being resistant to conventional therapy, including surgical procedures and chemotherapies, together with irradiation [2], and most patients relapse [3], surviving only between three and six months [4]. The 2016 WHO classification of CNS malignancies states that 50% of gliomas fall into the glioblastoma (GBM) category [5]. These tumors are highly invasive [6, 7], creating significant obstacles for treatment, leading to a median patient survival of between 14 and 15 months [8, 9]. Despite the increased use of electric field treatment, survival remains at less than 21 months [10]. Thus, new treatment strategies and targets and the development of new drugs are urgently needed.

Molecular-targeted therapy, encompassing immunotherapy, represents a pivotal advancement in managing malignant tumors, yet its application in glioma has encountered significant challenges. Most potential pharmaceutical interventions aimed at crucial pathways in GBM have demonstrated low efficacy throughout phase I and II clinical trials. Furthermore, the limited number of interventions that advanced to phase III primarily focus on modulating the tumor microenvironments (TMEs). These include Cilengitide, which blocks GBM integrin binding to the extracellular matrix and which failed phase III trials [11], and Bevacizumab, an anti-VEGF mouse-human chimeric antibody that blocks angiogenesis that resulted in some prolongation of progression-free but not overall survival [12]. Antibodies against the immune checkpoints PD-1/PD-L1 and CTLA-4 have proved unsuccessful in GBM treatment [13, 14]. The reasons for failure include TME complexity, high tumor heterogeneity/plasticity, and the blood–brain barrier preventing effective drug delivery. Thus, there is an urgent requirement for identifying molecules that can be targeted to prevent glioma progression.

The TCAF2 gene is located on chromosome 7 and contains three domains: the M60-like_N, Pep_M60_3, and peptidase_M60_domains, associated with metalloprotein kinase activity. The protein has been found to negatively regulate anion channel activity and positively modulate cellular migration [15]. Transient receptor potential (TRP) channels are members within the non-selective cationic channel superfamily and are involved in responses to exogenous stimuli and the pathogenesis of various tumors, including glioma [16]. The recruitment of TRPM8 to the cellular membrane and the facilitation of migration in prostate cancer cells is attributed to the actions of TCAF2, which operates in a TRPM8-dependent manner [15]. Studies have shown that TCAF2 is also involved in the malignant biological behavior of pancreatic cancer cells, affecting the prognosis of pancreatic cancer patients [17]. The latest research reports that TCAF2 can facilitate distant metastasis in colorectal cancer [18], and TCAF2 also involves glioma, which is associated with the immune microenvironment of glioma and promotes malignant progression [19]. However, research on TCAF2 in cancer remains limited, and its biological role and functions in glioma are not yet fully understood.

In the present study, the role of TCAF2 in glioma was investigated, finding that TCAF2 levels were positively correlated with significant clinicopathological features and patient prognosis. The results showed that upregulated TCAF2 enhanced migration/invasion properties in glioma cultures through an EMT-like process and STAT3 activation.

## Materials and methods

### Bioinformatics analysis

The TCGA and GTEx mRNA-seq data in perform Transcripts Per Million (TPM) format were downloaded from the UCSC XENA database (https://xenabrowser.net/datapages/) [20]. Then, TCAF2 levels were analyzed in glioma (*n* = 689) and normal tissue samples (*n* = 1157). The TCAF2 mRNA-seq data of glioma (*n* = 698) used in survival analysis and Cox regression analysis were downloaded from the TCGA database (https://portal.gdc.cancer.gov). TCGA supplementary clinical information included the WHO classification and IDH mutation together with 1p/19q co-deletion statuses from Ceccarelli et al. [21] and the prognostic data obtained from Liu et al. [22].

Subsequently, the relationship between TCAF2 expression levels and important clinical pathological factors was analyzed. Survival analysis was performed using the Kaplan–Meier (KM) method, with the median TCAF2 expression used as the cut-off value for high and low-expression groups. Univariate Cox regression analysis was conducted for TCAF2 expression and clinicopathological factors in the TCGA glioma, and significant variables identified through univariate Cox regression analysis (*p* ≤ 0.05) were further included in the multivariate Cox regression analysis. Further mRNA-seq and clinical data were obtained from the CGGA_mRNAseq_693 database to validate patient survival [23]. R "DESeq2" package identified differentially expressed genes (DEGs), and significant genes were analyzed by GSEA using the R "clusterProfiler" package [24, 25]. During bioinformatics analysis, NA values in each group were excluded.

### Clinical sample collection and tissue microarray (TMA)

TMA samples were extracted from patients designated for surgical resection within the Neurosurgery Department of Tianjin Medical University General Hospital between August 2011 and April 2017. Histopathologists performed pathological confirmation of the diagnosis according to the WHO criteria. All patients or relatives provided written informed consent. Such an investigation complied with the Declaration of Helsinki and the Ethics Committee of Tianjin Medical University General Hospital approvals. The TMA included 141 samples overall, of which 4 were normal, 21 were borderline, 25 were peritumoral, and 91 were tumor center samples (2 were WHO I, 18 were WHO II, 11 were WHO III, 60 were WHO IV).

### H&E staining immunohistochemistry (IHC)

IHC was conducted on TMAs of paraffin-embedded samples using the primary anti-TCAF2 antibody (1:50; ab2641227, Invitrogen, USA). A goat anti-rabbit IgG assay kit (ZSBG-Bio, China) was used for IHC marker assessment. Hematoxylin-nuclear staining was performed, while slides were imaged under a VANOX microscope (Olympus, Japan) and analyzed with Image J software to determine the integrated optical density (IOD). The IOD values were divided by the area of the target protein distribution to yield the average optical density (AOD). Paraffin-embedded tumor-bearing nude mice's brains and tumors were used for performing H&E staining as described previously [26].

### Cellular culturing

The human glioma cultures U251MG and U87MG were obtained through the Chinese Academy of Sciences Cell Bank (China), grown within DMEM (Gibco™, USA), and augmented through 10% fetal bovine serum (FBS). Glioma TJ905 culture was isolated from human GBM tissue and grown within F12 (Gibco™) + 10% FBS. Cultures were kept at 37 °C / 5% CO_2_.

### Lentivirus and plasmid transfection

We used the lentiviruses acquired from GeneChem, China, to establish TCAF2 knockdown, overexpression, and corresponding control cultures using three lines (U87MG, U251MG, and TJ905). The lentiviral transfection process was conducted according to the Protocol provided by GenChem. Cell selection was with 2.00 μg/ml puromycin. A STAT3-overexpressing plasmid was developed, employing a pcDNA3.1 vector. Plasmids were obtained from Hanbio (China) and underwent transient transfection into cultures through Lipofectamine 3000® (Invitrogen™).

### RNA collection / RT-qPCR

Overall RNA was extracted from tissues and cultures through TRIzol® (ThermoFisher Scientific™, USA) with 5 µg undergoing reverse transcription into cDNA through GoScript® Reverse Transcription System (Promega™, USA). RT-qPCR was performed per the previous Protocol [27] using Promega GoTaq qPCR Master Mix. GAPDH served as internal/normalization control. Primers (Genewiz, China) were as follows:

### TCAF2

Forward: 5'- AAAGTTGGGGTGAACACAAATCT-3'.

Reverse: 5'- CTTGTCACTGTACGCCTTGC-3'.

### GAPDH

Forward: 5'GGTGGTCTCCTCTGACTTCAACA-3'.

Reverse: 5’-GTTGCTGTAGCCAAATTCGTTGT-3'.

Results were calculated from the relative standard curve, followed by GAPDH normalization.

### CCK-8 assays

Following the directions provided by the manufacturer, cell viability was probed through CCK-8 (DOJINDO, China). Cultures were inoculated within 96-well plates (2.0 × 10^3^ cells / well) and grown under normal conditions for one, two, three, and four days. After the addition of CCK-8 reagent and incubation for 1 h, 450 nm-absorbance values were obtained employing a microplate reader (BioTek™, USA).

### Colony formation assays

Cells were cultured under standard conditions in 6-well plates (5.0 × 10^3^ cells / well) for 14 days. After rinsing in PBS, cultures underwent 4% paraformaldehyde-fixing, followed by 2.5% crystal violet-staining steps. After imaging, the number of clone formations is counted using ImageJ software. Colony formation was determined as the ratio between the colony numbers and the number of seeded cells.

### Cell scratch assays

Cells (1.5 × 10^5^ cells/well) were grown under normal conditions in 6-well plates until reaching approximately 80% confluence. After several washes in PBS, a 200-μl pipette tip scratched three straight lines per well within the culture monolayer, followed by PBS-rinsing (thrice) to remove floating cells and incubated within serum-free media (24 or 48 h). Scratch areas were examined and imaged at 0, 24, and 48 h.

Subsequently, the scratch area at each corresponding time point was calculated using ImageJ software, Cell migration rate = (0 h scratch area−48 h scratch area)/0 h scratch area × 100%.

### Transwell assay

Concerning probing of migratory property, cultures (1.0 × 10^4^ cells / well) within serum-free DMEM or DMEM/F12 were placed within the upper chamber (Corning, USA). Media containing 10% were introduced within lower-chamber. Concerning invasive properties, the chamber was Matrigel-coated, and cells (5.0 × 10^4^ cells/well) in serum-free DMEM were added and incubated (one day). The non-invasive cells within the upper chamber were then collected with a cotton swab. The invasive cells were fixed using 4% Paraformaldehyde, stained with a 2.5% solution of crystal Violet Stain, and imaged as above. Image J software quantified cellular numbers within the lower surfaces of such filters.

### Western blotting

Cells underwent RIPA buffer lysis (Solarbio™) having 1:100 PMSF, 1:100 phosphatase inhibitors (Solarbio) and 1:100 protease inhibitor mixture (Solarbio). Proteomic levels were determined through the BCA kit (Solarbio). Thirty micrograms of protein was segregated on SDS-PAGE and blotted onto PVDF (Millipore, USA), followed by a 5% skimmed milk block. Membranes were probed overnight at 4℃ with primary antibodies followed by secondary antibodies (1:3000; ZB-2301, ZB-2305, ZSGB-BIO, China) (one hour / ambient temperature). Proteomic bands were visualized through GBOX® (Syngene™ Company, UK) and a chemiluminescent HRP substrate (Millipore™). Primary antibodies (all 1:1000 dilutions) were against TCAF2 (Invitrogen), E-Cadherin (E-Ca), N-Cadherin (N-Ca), Vimentin, Snail, and p-STAT3, all from Cell Signaling Technology (USA), STAT3 was procured through ABclonal® (China), β-actin procured through ZSGB-BIO™ (China).

### Mouse xenograft models

All animal protocols received approval from the Ethical Committee of Tianjin Medical University General Hospital. BALB/c-nu female nude mice, aged 6 weeks with a weight of approximately 16–18 g, were randomly divided into empty Vector, OE-TCAF2, sh-NC, and sh-TCAF2, with 5 mice per group. A mouse model with intracranial tumors was established by stereotactically implanting 0.5 × 10^5^ U251MG cells infected with lenti-empty vector, lenti-OE-TCAF2, lenti-sh-NC, or lenti-sh-TCAF2 into the respective groups using cranial guide screws (RWD Life Science, China) [26]. Subsequently, the mice were transferred to isolators and fed with pathogen-free feed. The tumor growth was analyzed weekly through bioluminescence imaging beginning upon day 7 post-injection through IVIS Spectrum Live Imaging System (Perkin Elmer™, USA). Up to death, the survival of mice in both groups was assessed daily. After mice died, their brains and tumors were extracted and fixed in 10% formalin, then embedded in paraffin for subsequent H&E staining.

### Statistics

All experiments were conducted a minimum of three times, and datasets were analyzed through SPSS 20®. Quantitative datasets reflected mean ± SD while variations across two cohorts were comparatively analyzed through unpaired t-tests with two-tailed *p*-values < 0.05 deemed to confer statistical significance. Murine survival was analyzed through the survival package in R (4.2.1) to determine the proportional hazards and visualized using the survminer and ggplot2 packages.

## Results

### TCAF2 is upregulated in glioma and is associated with prognosis

**Fig. 1:**
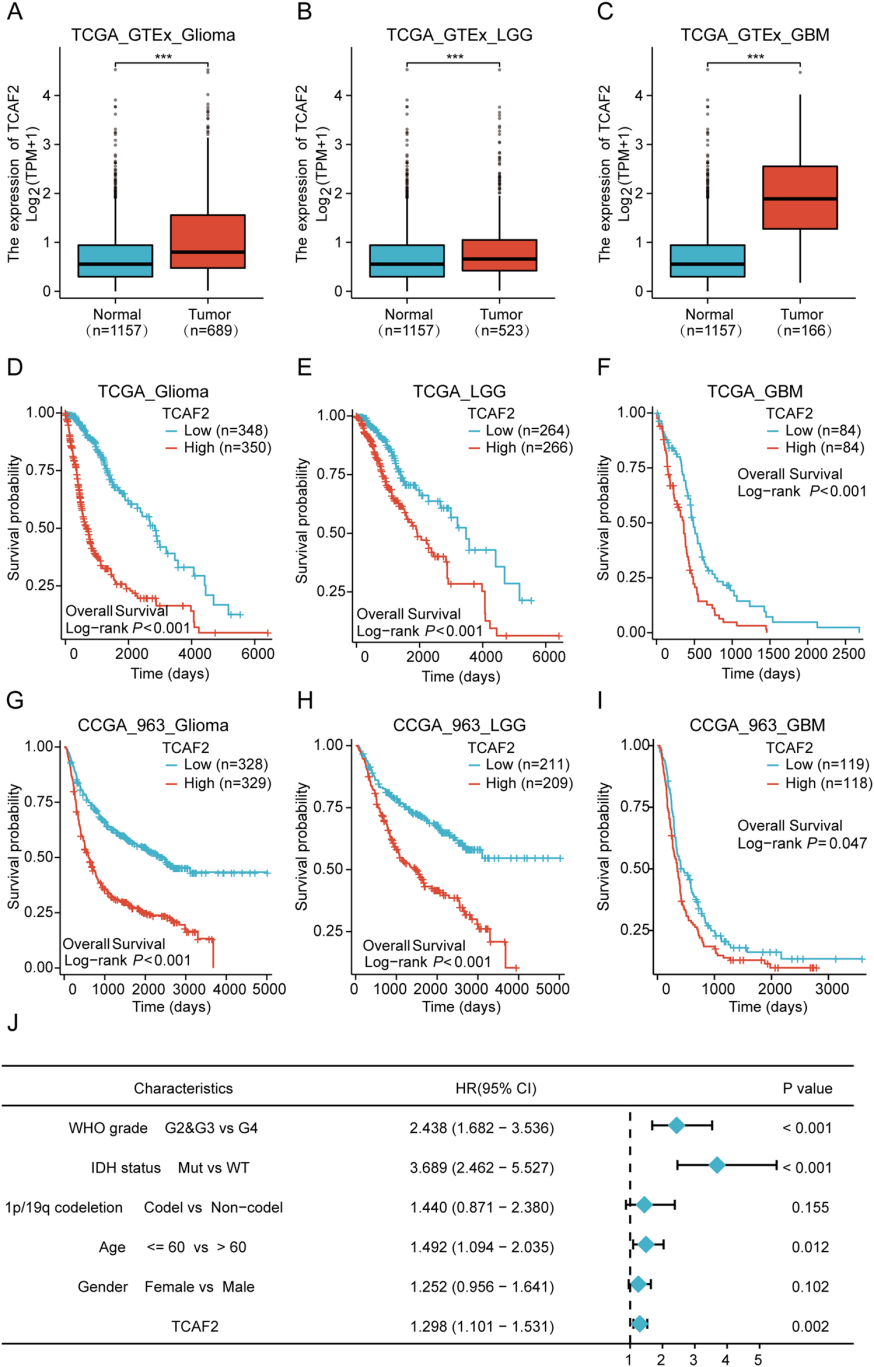
The expression of TCAF2 in glioma and its relationship with glioma prognosis. **A**–**C** TCAF2 levels in glioma (*n* = 689), LGG (*n* = 523), GBM (*n* = 166), and normal tissues (*n* = 1157) in UCSC data. **D**–**F** Survival analysis of glioma (*n* = 698), LGG (*n* = 530), and GBM (*n* = 168) in the TCGA database after grouping them based on the median value of TCAF2 expression. **G**–**I** Survival analysis of glioma (*n* = 657), LGG (*n* = 420), and GBM (*n* = 237) patients in CCGA-693 data after grouping them based on the median value of TCAF2 expression. **J** Multivariate Cox analysis of clinicopathological factors and TCAF2 levels in glioma in TCGA data (***p < 0.001)

Analysis of Toil-processed the TPM-format TCGA and GTEx RNA-seq data from UCSC XENA-dataset outcomes showed TCAF2 expression within glioma, GBM, and low-grade glioma (LGG) was considerably upregulated in comparison with healthy brain tissue (Fig. 1A-C). Further probing for associations across TCAF2 levels and overall survival (OS) within TCGA data indicated that regardless of whether the diagnosis was a glioma, LGG, or GBM, cases having upregulated TCAF2 expression had shorter OS in comparison with patients having lower TCAF2 expression (Fig. 1D-F). CGGA-693 provided additional confirmation, with consistent results for the findings from the TCGA data (Fig. 1G-I). Univariate and multivariate Cox analyses were conducted to assess the association between TCAF2 expression and various clinicopathological characteristics in the TCGA datasets to identify the independent prognostic markers (Figure S1). Dataset outcomes revealed that TCAF2 level, age, WHO grade, IDH, and 1p/19q status were independent prognostic factors for glioma (Fig. 1J).

### TCAF2 levels are associated with significant clinicopathological features of glioma

**Fig. 2:**
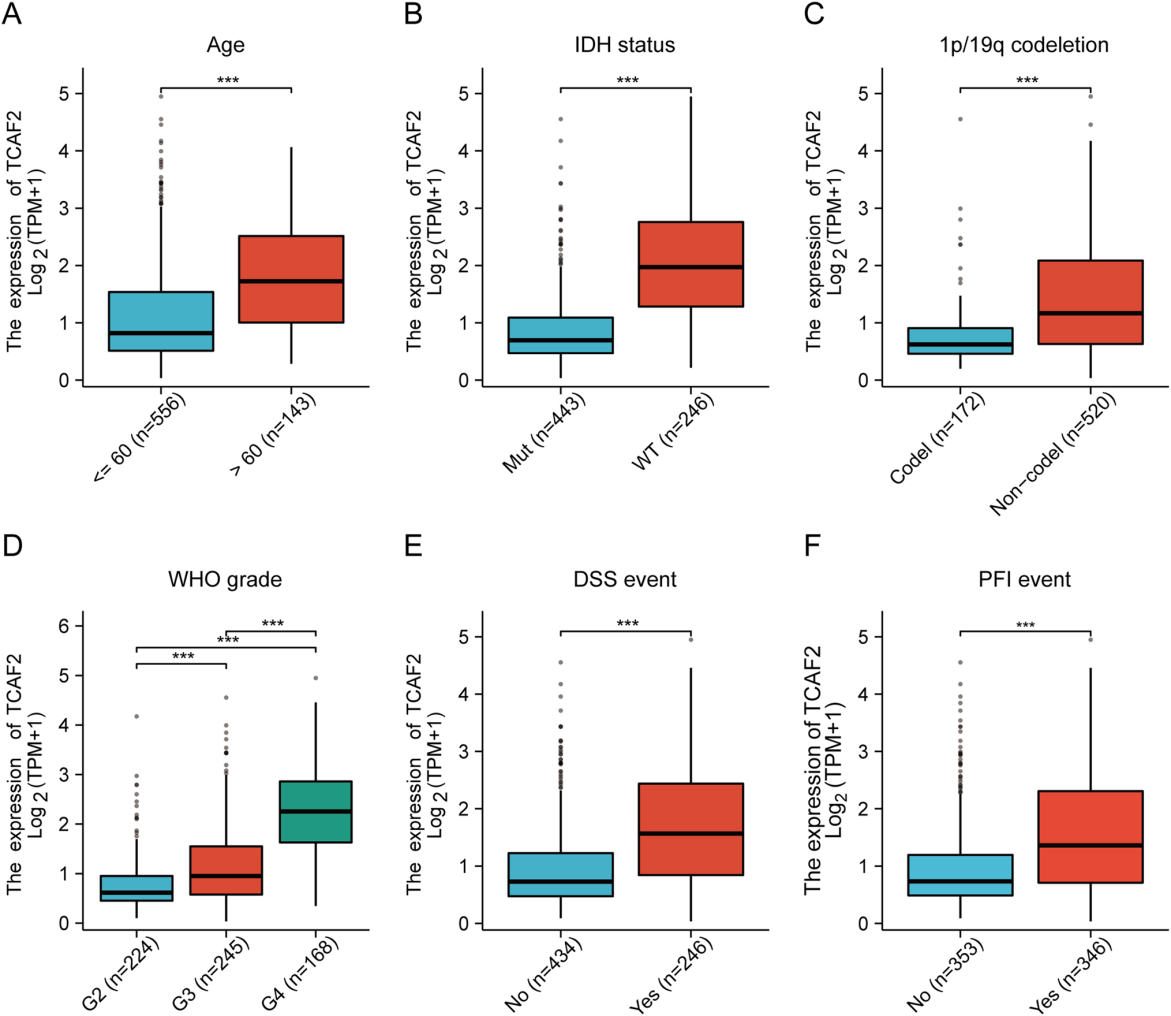
Relationship between TCAF2 levels and clinicopathological features of glioma patients in TCGA data. **A**–**F** Clinicopathological features including age (age < 60 *n* = 556, age > 60 *n* = 143), IDH status (mutation *n* = 443, wild type *n* = 246), 1p/19q co-deletion status (co-deletion *n* = 172, non-co-deletion *n* = 520), WHO grade (grade 2 *n* = 224, grade 3 *n* = 245, grade 4 *n* = 168), DSS (no *n* = 434, yes *n* = 246), and PFI (no *n* = 353, yes = 346) (***p < 0.001)

Analysis for associations across TCAF2 expression profiles and patient characteristics within TCGA data indicated significant associations between the TCAF2 level and age, WHO grade, IDH and 1p/19q status, progression-free interval (PFI), and disease-specific survival (DSS). Furthermore, in elderly cases (Fig. 2A), IDH wild-type (IDH-WT) patients (Fig. 2B), and patients with 1p/19q non-co-deletions (Fig. 2C), as well as in the PFI and DSS event groups (Figs. 2E-F), TCAF2 levels were markedly elevated. TCAF2 levels were also increased significantly in higher-grade tumors (Fig. 2D). The expression level of TCAF2 was found to be significantly positively correlated with the expression of IDH1 (*R* = 0.484, *p* < 0.001) (Figure S2A). After classifying gliomas in the TCGA database according to the WHO 5th edition classification of central nervous system tumors, it was observed that the expression of TCAF2 increased with tumor grade in the IDH mutation group. In contrast, its expression level was significantly higher in the IDH-WT group than in other groups (Figure S2B). Clinical grouping based on IDH status revealed that patients with high TCAF2 expression had a poorer prognosis, regardless of whether they belonged to the IDH mutation or IDH-WT group (Figure S2C-D).

### TCAF2 protein levels are associated with tumor grade and specimen location

**Fig. 3:**
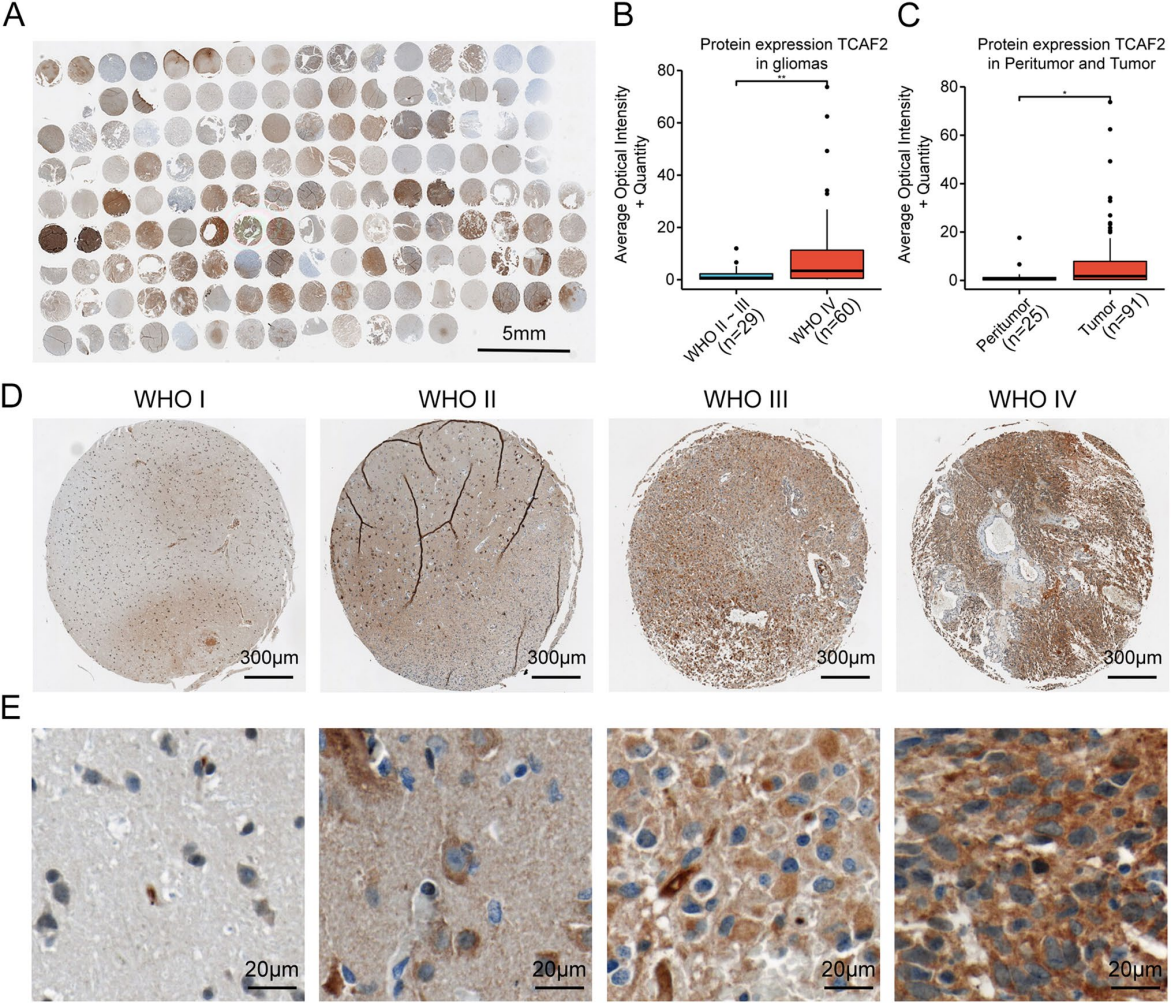
Immunohistochemical staining of glioma tissue microarray. **A** Expression of TCAF2 in TMA samples. **B**–**C** Higher TCAF2 levels were seen in GBM (*n* = 60) relative to LGG (*n* = 29) and in tumor centers (*n* = 91) relative to the periphery (*n* = 25). **D** Higher levels of TCAF2 with increased tumor grade (D, E) (*p < 0.05, **p < 0.01)

For further verification of TCAF2 protein levels in glioma tissues, specimens were collected at our institution, made into TMAs, and stained by IHC. TCAF2 was strongly expressed within glioma and peritumoral tissue (Fig. 3A). Figures 3D and 3E show the IHC staining of samples from tumors of different WHO grades. It was found that TCAF2 protein levels were markedly elevated in GBM samples compared with LGG specimens (Fig. 3B), and protein levels in samples taken from the tumor-mass center were considerably upregulated in comparison with counterpart levels within the tumor periphery (Fig. 3C). These results were in agreement with those on the TCGA data.

### Overexpression of TCAF2 promotes migration/invasion properties within glioma cells

**Fig. 4:**
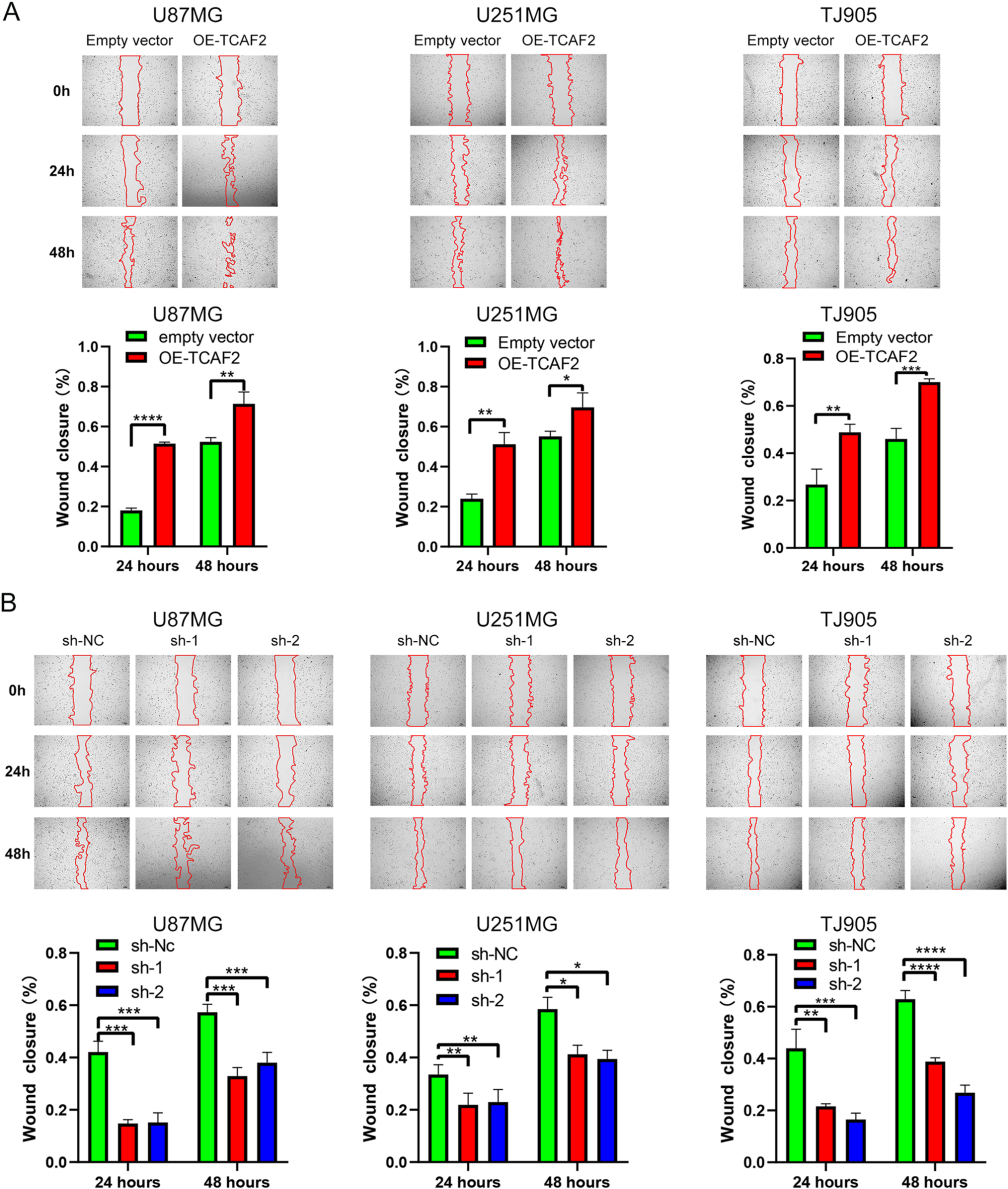
TCAF2 overexpression promoted scratch healing. **A** Overexpression of TCAF2 (OE-TCAF2) promoted scratch healing in U87 MG, U251 MG, and TJ905 cells at 24 h and 48 h. **B** TCAF2 knockdown (shRNA-TCAF2-1, sh-1; shRNA-TCAF2-2, sh-2) in U87 MG, U251 MG, and TJ905 cultures inhibited glioma cell migration at 24 h and 48 h. Wavy red lines represent wound edges (*p < 0.05, **p < 0.01, ***p < 0.001, ****p < 0.0001)

**Fig. 5:**
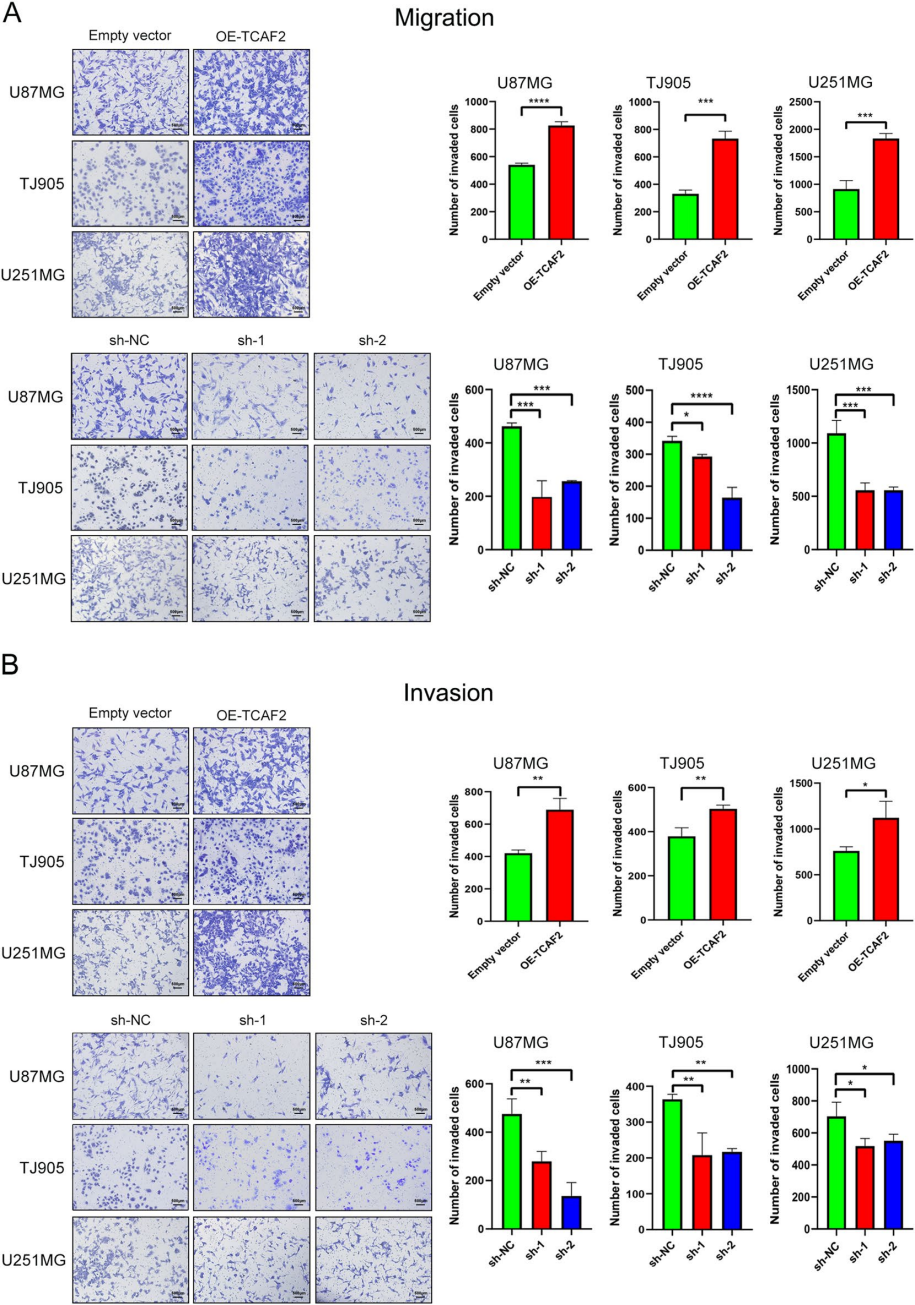
TCAF2 overexpression promoted migratory/invasive properties of glioma cells. **A** Migration assay showed that TCAF2 overexpression (OE-TCAF2) promoted migration of U87 MG, U251 MG, and TJ905 cells, whereas knockdown of TCAF2 (shRNA-TCAF2-1, sh-1; shRNA-TCAF2-2, sh-2) inhibited migration. **B** Invasion assay showed that overexpression of TCAF2 promoted invasion of U87 MG, U251 MG, and TJ905 cells, whereas knockdown of TCAF2 inhibited invasion (*p < 0.05, **p < 0.01, ***p < 0.001, ****p < 0.0001)

TCAF2 overexpression (OE-TCAF2) and knockdown (shRNA-TCAF2-1, sh-1; shRNA-TCAF2-2, sh-2) effectiveness within three cultures were examined through both RT-qPCR and western blotting (Figure S3A-C). Proliferation was assessed by colony formation and CCK8 assays. It was found that neither overexpression nor knockdown of TCAF2 significantly affected glioma cell proliferation (Figure S4A, B and Figure S5A-C). In the scratch and migration assay, TCAF2 overexpression was found to promote migration, while knockdown had the reverse effect (Fig. 4A, B). Similar results were obtained in the Transwell assays (Fig. 5A). Assessment of cell invasion in the Matrigel-coated Transwell chamber indicated that invasion was enhanced by TCAF2 overexpression and correspondingly reduced by TCAF2 knockdown (Fig. 5B). Thus, the results of the scratch and Transwell assays were consistent.

### TCAF2 promotes migration/invasion properties through EMT-like processes stimulation

**Fig. 6:**
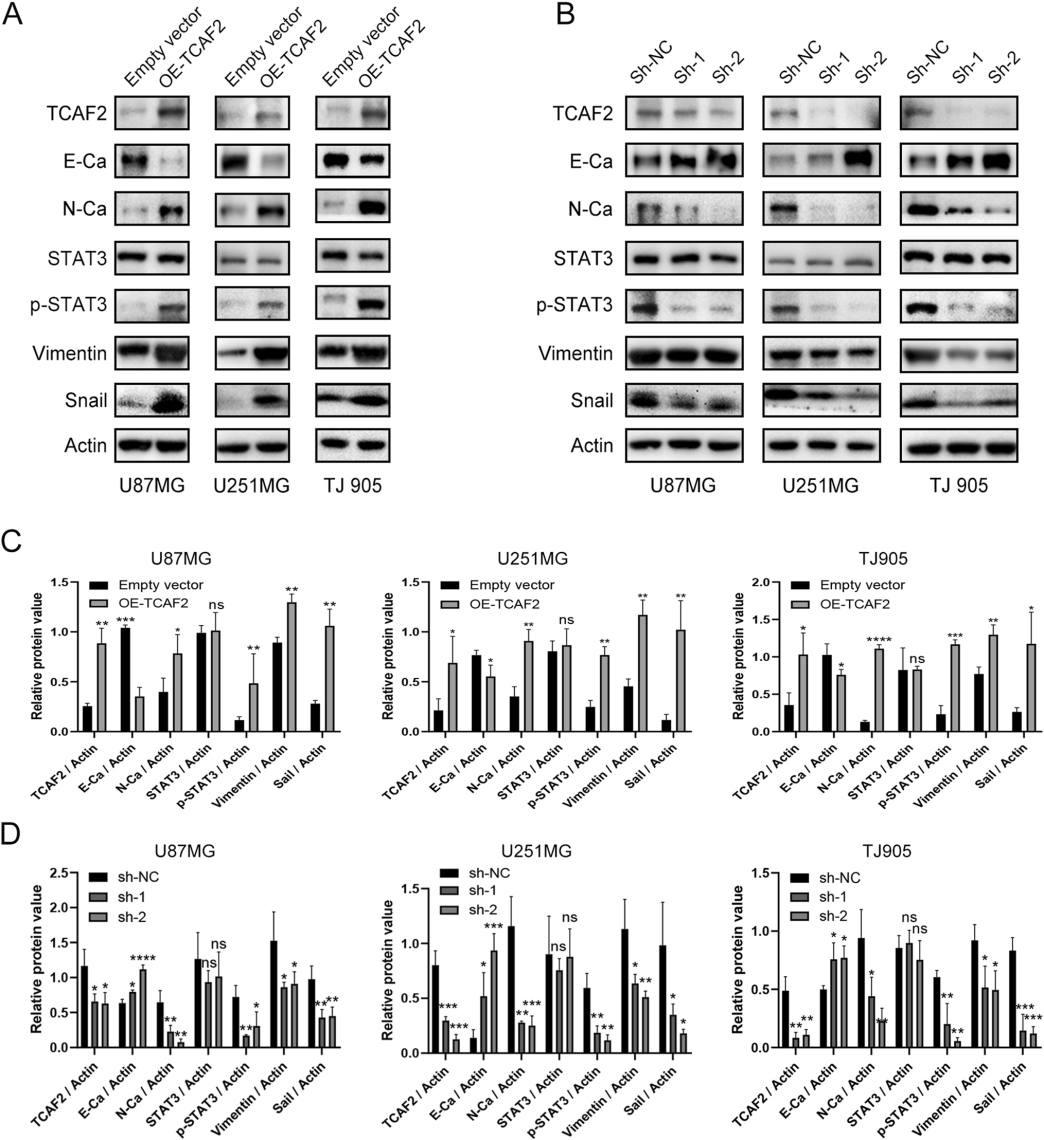
TCAF2 promotes the migratory/invasive properties of glioma cells by regulating the EMT and is correlated with STAT3 signaling in glioma cells. **A**–**B** Western blotting showing changes in TCAF2 and EMT markers (E-cadherin, N-cadherin, Vimentin, and Snail), STAT3 and p-STAT3 expression in U87MG, U251MG, and TJ905 glioma cells after overexpression and knockdown of TCAF2. **C**–**D** The relative TCAF2, E-Cadherin, N-Cadherin, Vimentin, Snail, STAT3, and p-STAT3 levels close to β-actin (ns *p* > 0.05, *p < 0.05, **p < 0.01, ***p < 0.001, ****p < 0.0001)

GSEA analysis of the HALLMARK gene set according to the Log2FoldChange values found that genes associated with TCAF2 were strongly enriched in the epithelial–mesenchymal transition (EMT) (NES = 2.383, adjust *p*-value [*p*. adj] < 0.0001) (Figure S6). Western blotting was then used to confirm these findings, showing significant changes in the levels of EMT markers after TCAF2 overexpression and knockdown. E-Ca levels were reduced in all cells overexpressing TCAF2, while those of N-Ca, Vimentin, and Snail were increased (Fig. 6A, C). In contrast, TCAF2 knockdown elevated E-Ca levels while reducing those of N-Ca, Vimentin, and Snail (Fig. 6B, D). Such results infer that TCAF2 overexpression enhances glioma cell motility by stimulating EMT-like processes.

### TCAF2 promotes migratory/invasive properties by activation of STAT3

**Fig. 7:**
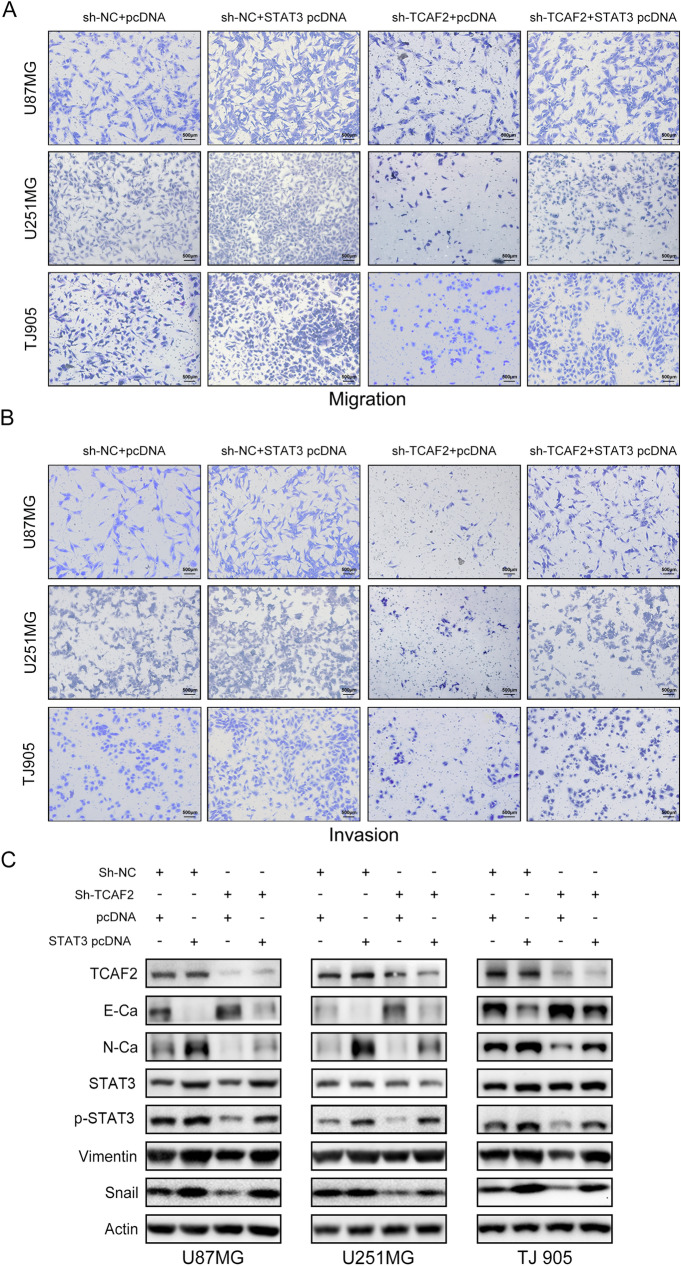
TCAF2 mediated migration/invasion properties of glioma cells by activating STAT3 signaling. **A**–**B** Transwell assay showing that STAT3 overexpression counteracted the effects of TCAF2 knockdown on migration and invasion. **C** Western blotting results show TCAF2, STAT3, p-STAT3, E-Ca, N-Ca, Vimentin, and Snail levels in the three glioma cells

GSEA was performed to understand the molecular mechanism of TCAF2 further, showing enrichment of TCAF2-associated genes within the IL6-JAK-STAT3 pathway (NES = 2.249, adjust p-value [p. adj] < 0.0001) (Figure S7). Western blotting evaluated the levels of significant components of STAT3 signaling, specifically STAT3 and p-STAT3. Significantly upregulated p-STAT3 was noted within cells overexpressing TCAF2, while the levels of STAT3 were not altered (Fig. 6A, 6C). In contrast, p-STAT3 levels were significantly lower after TCAF2 knockdown, seen in all three cultures (Fig. 6B, 6D). Further experiments using STAT3 overexpression showed that this counteracted the effects of TCAF2 knockdown (Fig. 7A, B). As demonstrated by western blotting, STAT3 overexpression also reduced the levels of E-cadherin while increasing those of N-cadherin, Vimentin, and Snail in TCAF2-knockdown cells (Fig. 7C). These findings indicate that TCAF2 promoted cellular migration, invasion, and EMT-like processes by activating STAT3.

### TCAF2 overexpression decreases survival in vivo

**Fig. 8:**
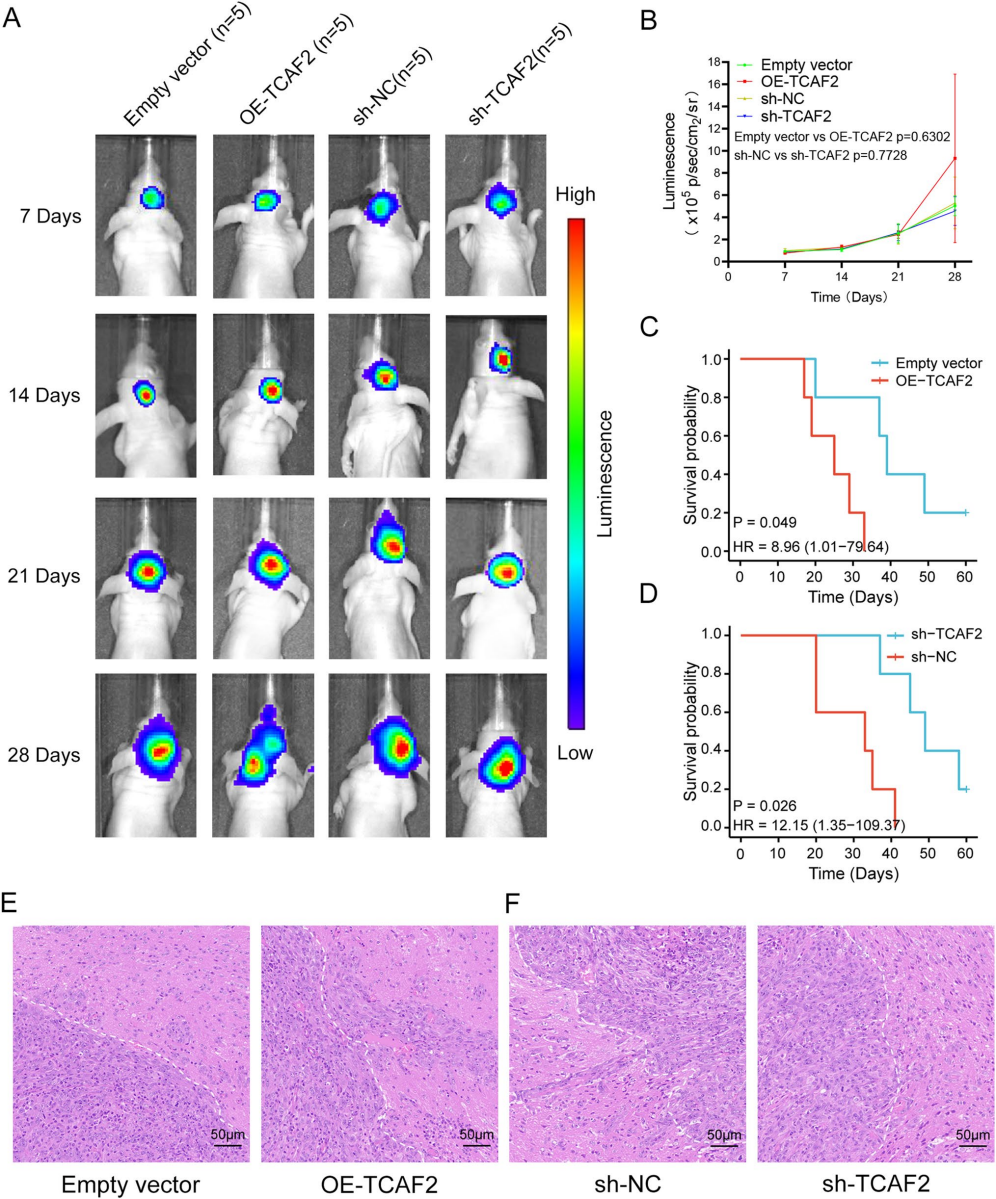
TCAF2 overexpression is linked to reduced overall survival in vivo. **A** Bioluminescence imaging of tumor growth in U251MG intracranial xenograft mouse (*n* = 5 per group) each week beginning on day 7 after implantation. **B** Signal intensities were quantified on days 7, 14, 21, and 28 after implantation. **C**–**D** Kaplan–Meier survival curves indicating the percentage survival of mice. **E–F** H&E staining was used to observe the tumor boundaries of tumor-bearing mice, and the tumors of U251MG-OE-TCAF2 mice showed invasive boundaries, whereas the tumors of U251MG-sh-TCAF2 mice showed smooth boundaries

TCAF2 functions were further evaluated in a xenograft mouse model. The mice were monitored by Luminescence weekly for four weeks (Fig. 8A). Compared to the control cohort, neither overexpression nor TCAF2 knockdown considerably affected tumor growth (Fig. 8B). Kaplan–Meier analysis showed that U251MG-OE-TCAF2 tumor-bearing mice survived for shorter periods than those in the empty vector group (Fig. 8C). In contrast, mice injected with U251MG-sh-TCAF2 cells survived longer than the controls (Fig. 8D). The H&E staining analysis revealed that the tumors present in U251MG-OE-TCAF2 tumor-bearing mice exhibited an infiltrative border (Fig. 8E). In contrast, the tumors detected in U251MG-sh-TCAF2 animals presented a smooth border (Fig. 8F). The results of this study indicate that the overexpression of TCAF2 leads to an increase in the migratory and invasive capabilities of glioma cells, as well as a decrease in survival rates among xenograft mice. These findings align with the outcomes observed in vitro.

## Discussion

Glioma is a common and deadly brain tumor with poor outcomes. Treatment of glioma to improve the prognosis is hugely challenging. Over the past several decades, studies of glioma biology have found various molecular changes within grade II, III, and IV gliomas, two of which are particularly important because they are prevalent, present in early-stage gliomas, or are closely connected to overall survival. The first is 1p/19q co-deletion, linked to oligodendrocyte tissue type and sensitivity to alkylating drug treatment [28, 29]. The second is a mutation in the IDH genes, IDH1 or IDH2, which is not specific to particular histopathological glioma subtypes but is linked with cellular metabolism [5, 30]. These factors significantly affect glioma grading, stratification, diagnosis, and management.

Interestingly, we observed similar results when conducting extensive data bioinformatics analysis. We observed broad expression and significant enrichment of TCAF2 in both LGG and GBM samples, with a significant association between increased TCAF2 levels and reduced patient survival (Fig. 1A-I). Numerous studies have found that IDH status [31, 32], WHO grade [33], and older age [34] are reliable factors for predicting glioma prognosis and are widely applied clinically. Regression analyses showed that TCAF2 could independently predict glioma prognosis (Fig. 1J), similar to IDH status, WHO grade, and older age. Regarding the relationship between TCAF2 and clinicopathological factors, significant associations were observed between TCAF2 levels and the 1p/19q and IDH status, age, WHO grade, DSS, and PFI (Fig. 2, S2). Analysis of 137 glioma specimens by IHC produced consistent results (Fig. 3). Thus, it can be speculated that TCAF2 is closely associated with glioma pathogenesis.

Earlier studies reported that TCAF2, as a chaperone of TMPR8, is implicated in invasiveness/migration for both pancreatic and prostate cancer [15, 17]. Recent research reports that TCAF2 in peritumoral stromal cells promotes liver metastasis in colorectal cancer by inhibiting the cold-sensing TRPM8 channel [18]. Additionally, a recent study reported that TCAF2 is associated with the immune microenvironment of the glioma, promoting its onset and impairing prognosis [19]. TCAF2 is a membrane protein containing four domains. Three are associated with metallopeptidase activity, functioning mainly in mediating cell–cell adhesion, which may explain the association with migration and invasion properties in glioma. Overexpression of TCAF2 significantly promoted both migratory/invasive properties in glioma cells (Figs. 4,5,8E). GSEA suggested marked enrichment of TCAF2-related DEGs in the EMT, which ranked top in all the enrichment results (Figure S6), and the levels of EMT markers altered accordingly. Our findings are consistent with both the bioinformatics analysis and the study conducted by Li et al. and provide additional insights into the mechanisms by which TCAF2 promotes malignant progression in glioma.

The JAK-STAT pathway represents a classical pathway involved in STAT-related signaling and consists essentially of a receptor, the JAK tyrosine kinase, together with the transcription factor STAT. The pathway contributes significantly to numerous cellular processes. Growth factors and cytokines interact and form complexes with cell-surface receptors, such as IL6/IL6R, EGF/EGFR, and STAT3, activating it. Tyrosine phosphorylation of STAT3 leads to dimerization followed by nuclear translocation, which regulates EMT-associated genes' transcription, such as Twist, Snail, and Slug [35]. The JAK/STAT3/Slug, JAK/STAT3/Snail, and other pathways activate EMT-related transcription factors that modulate the EMT and thus promote tumorigenesis [36, 37]. Promoting the EMT-like processes in high-grade glioma has been linked to an aggressive phenotype and resistance to treatment, resulting in poor patient outcomes [38, 39]. GSEA indicated marked enrichment of the TCAF2-associated DEGs in IL6-JAK-STAT3 signaling (Figure S7), and TCAF2 overexpression downregulated E-cadherin while upregulating the levels of N-cadherin, Snail, and Vimentin, and promoting the EMT-like processes (Fig. 6). It was also found that TCAF2 knockdown reduced the levels of p-STAT3 (Fig. 6). Such dataset outcomes are consistent with numerous reports that STAT3 influences EMT biomarker levels in tumor cells [40, 41]. Notably, STAT3 overexpression rescued the influence of TCAF2 knockdown on migration/invasion, together with EMT-like processes, suggesting that TCAF2 promotes glioma cell migratory/invasive properties through activating STAT3 signaling (Fig. 7).

Nevertheless, this preliminary investigation of TCAF2 in glioma has several limitations. Firstly, additional experiments are required to observe tumors' invasive behavior in vivo accurately. Secondly, the mechanism underlying TCAF2 mediation of the enhancement of invasion and migration of glioma cells remains unclear, and more studies are needed to clarify the downstream mechanism of TCAF2 action in glioma. Lastly, it is necessary to design drugs targeting TCAF2 to elucidate the mechanism across multiple study levels.

In essence, this study investigated the role of TCAF2 in glioma. TCAF2 was positively related to tumor grade, 1p/19q, IDH status, and poor outcome. In vitro and in vivo experiments demonstrated that TCAF2 promotes the malignant progression of glioma cells, proposing that TCAF2 may be a valuable target for treating glioma.

### Supplementary Information

Below is the link to the electronic supplementary material.

Supplementary file1 (DOCX 2986 kb)

## Data Availability

The data that support the findings of this study are available in the figures and the supplementary material of this article.
