# Supplementary material for: TCAF2 drives glioma cellular migratory/invasion properties through STAT3 signaling
Source: Mol Cell Biochem. 2023 Nov 29;479(7):1801–15. doi: 10.1007/s11010-023-04891-0 (PMC11255011; doi:10.1007/s11010-023-04891-0)

**Figure S1.** Univariate Cox analysis of clinicopathological factors and TCAF2 expression levels of glioma in TCGA database.

**Figure S2.** **A.** The expression of TCAF2 was significantly positively correlated with the expression of IDH1. **B.** TCAF2 expression increased with tumor grade in the IDH-Mut group (Including WHO II n=203, WHO III n=175, WHO IV n=12), with the highest expression observed in the IDH-WT group (n=238). **C-D.** Regardless of IDH status (IDH-Mut or IDH-WT), patients with high TCAF2 expression had worse overall survival (*p<0.05, **p<0.01, ***p<0.001).

**Figure S3. A-C.** RT-PCR and western blotting results showed the transfection efficiency of lentiviral overexpression (OE-TCAF2) and knockdown (shRNA-TCAF2-1, sh-1; shRNA-TCAF2-2, sh-2) expression of TCAF2 in U87MG, U251MG, and TJ905 glioma cultures. (**p<0.01, ***p<0.001).

**Figure S4. A-B.** The results of CCK-8 showed that the overexpression (OE-TCAF2) and knockdown (shRNA-TCAF2-1, sh-1; shRNA-TCAF2-2, sh-2) expression of TCAF2 had no significant effect on the proliferation of U87MG, U251MG, and TJ905 glioma cells.

**Figure S5. A-C.** Colony formation assay results showed that the overexpression (OE-TCAF2) and knockdown (shRNA-TCAF2-1, sh-1; shRNA-TCAF2-2, sh-2) expression of TCAF2 had no significant effect on the proliferation of U87MG, U251MG and TJ905 glioma cells (ns p>0.05).

**Figure S6.** GSEA enrichment analysis shows that TCAF2-associated genes are highly enriched in the process of epithelial–mesenchymal transition (NES=2.383, adjust p-value (p. adj) <0.001).

**Figure S7.** GSEA enrichment analysis shows that TCAF2-associated genes are highly enriched in the IL6-JAK-STAT3 signaling pathway (NES=2.249, adjust p-value (p. adj) <0.001).


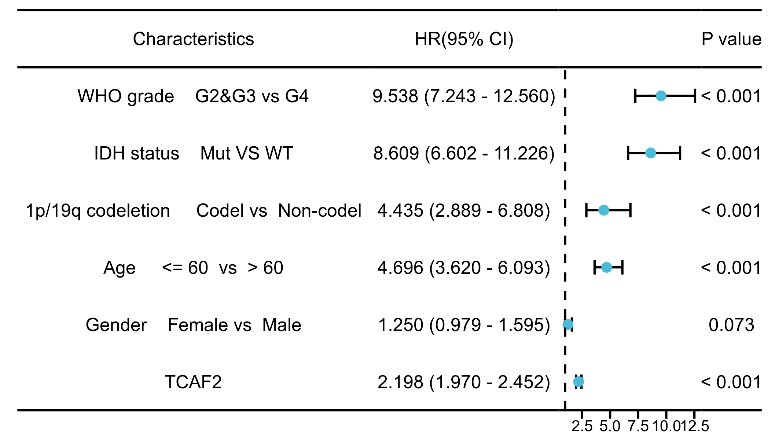


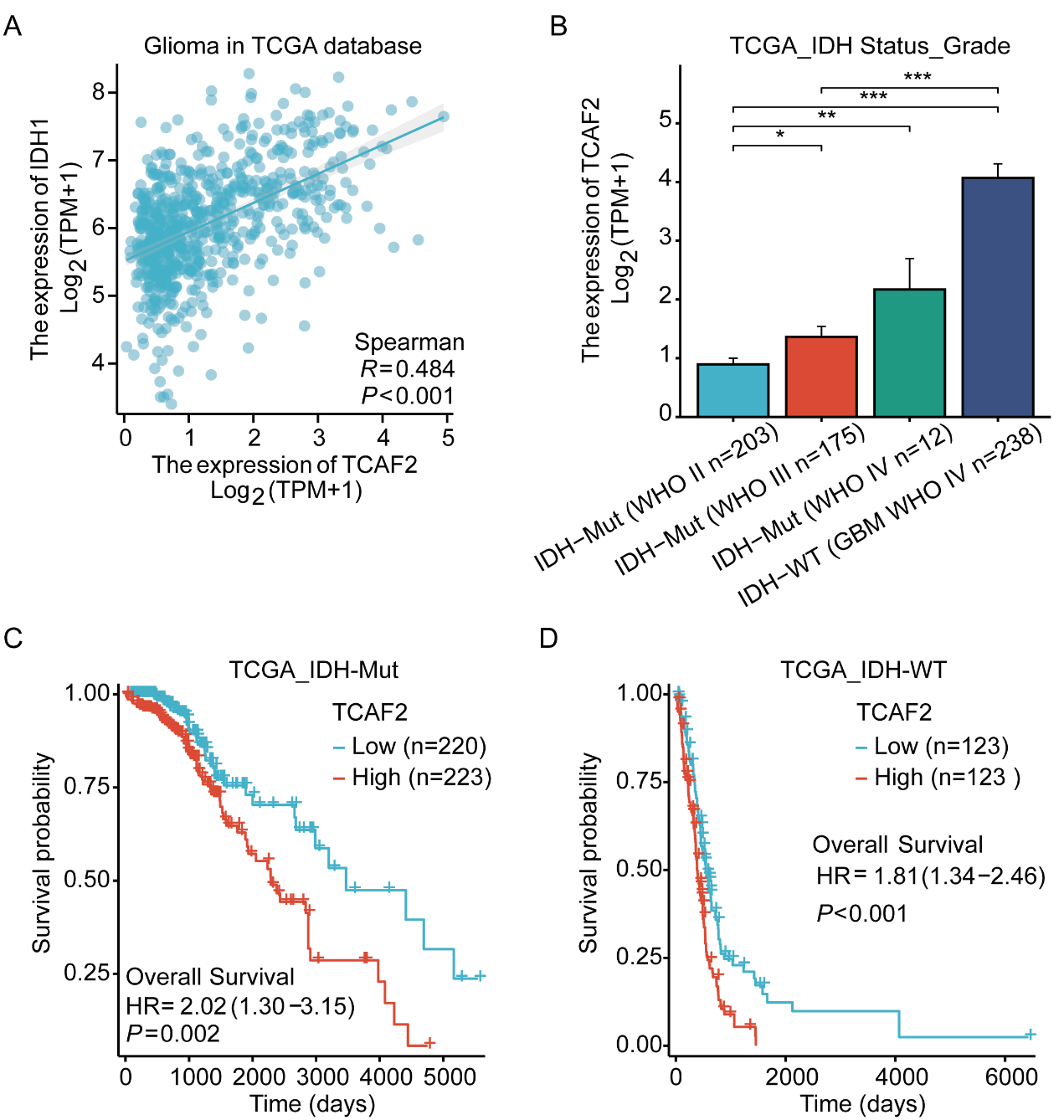


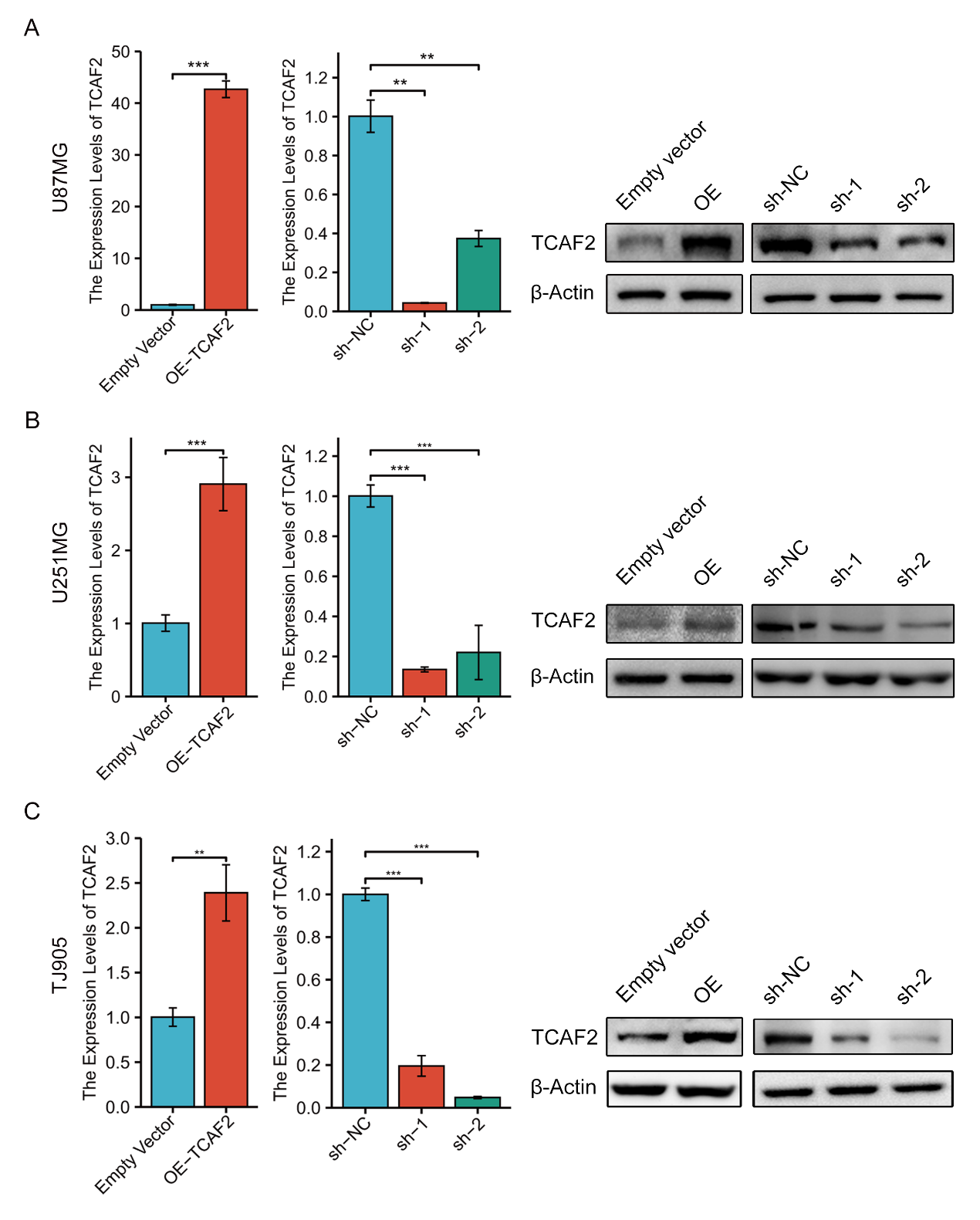


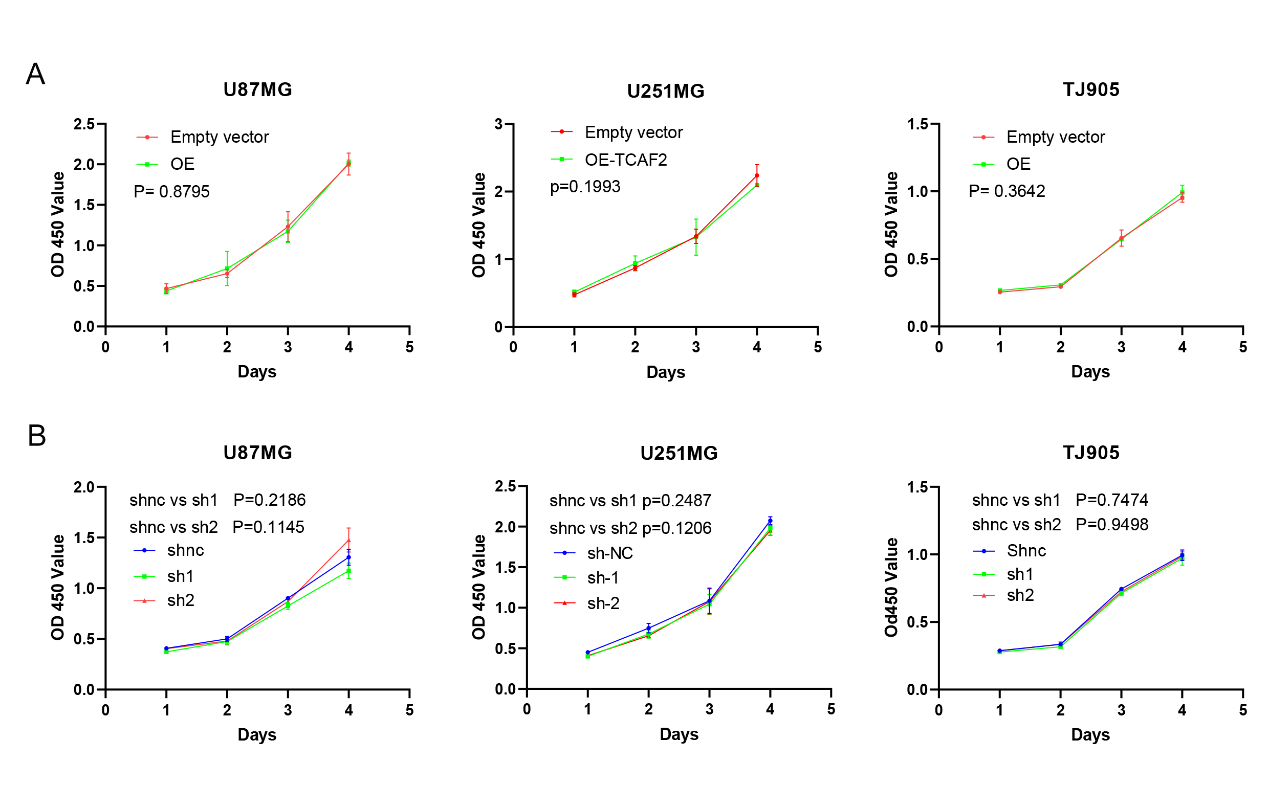


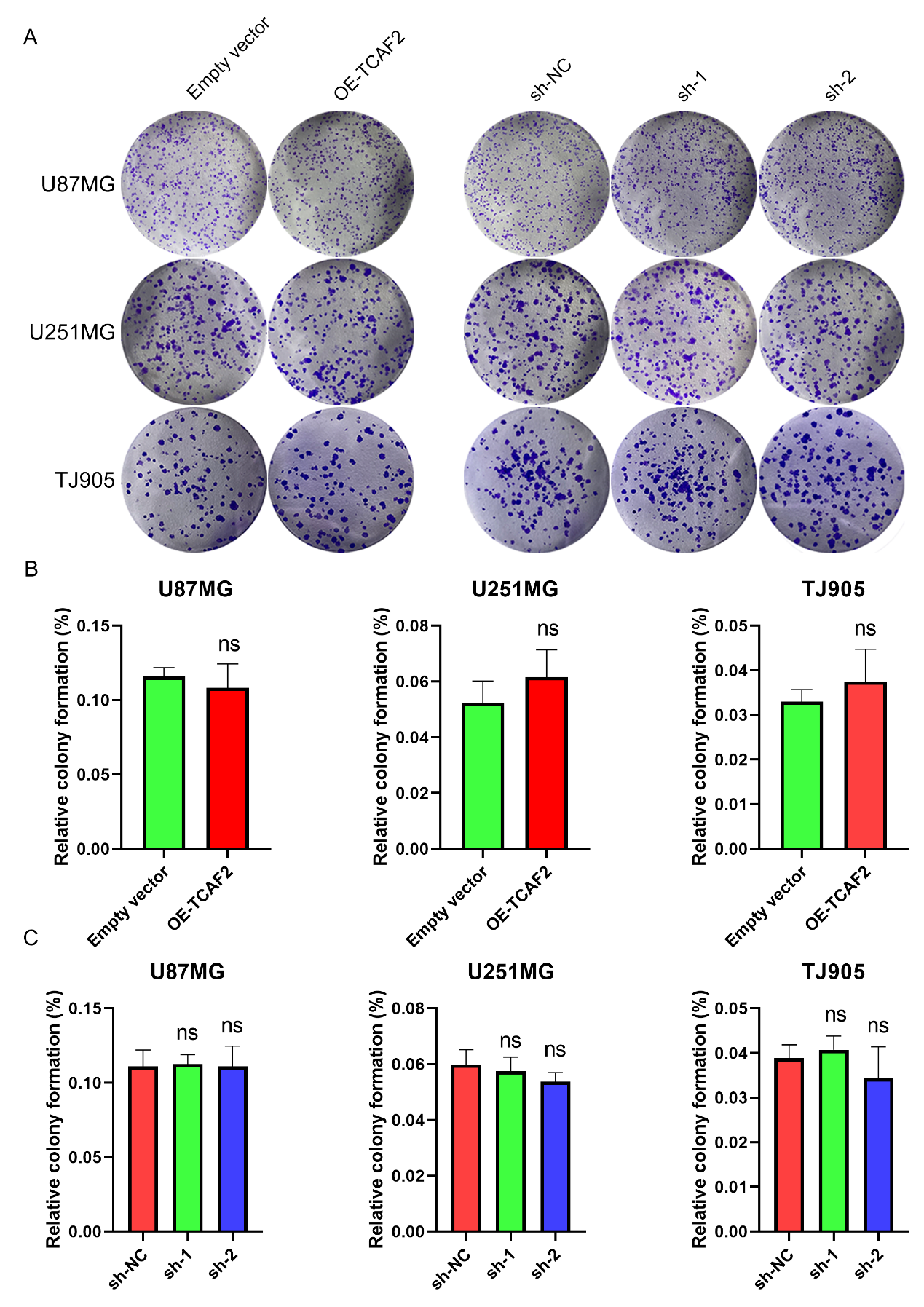


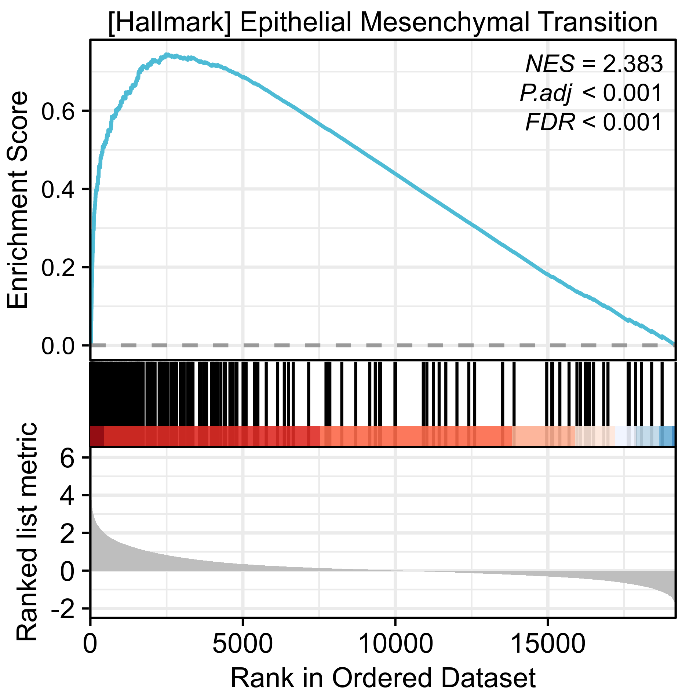


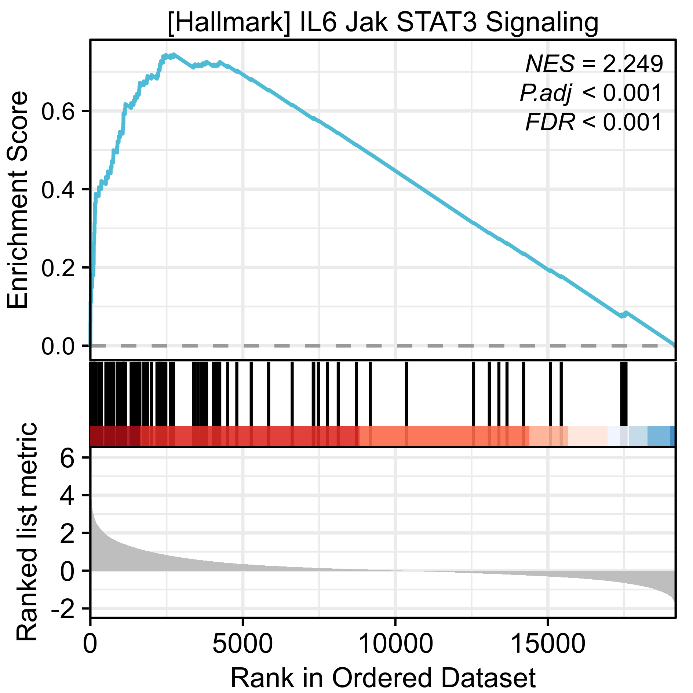

Supplement: Supplementary file 1 — Supplementary file1 (DOCX 2986 kb) [file 11010_2023_4891_MOESM1_ESM.docx]
